# Factors Affecting the Help-Seeking Behaviours of Patients with Schizophrenia in Rural Cambodia

**DOI:** 10.1155/2020/8065058

**Published:** 2020-06-05

**Authors:** Vantheara Oum, Yurie Kobashi, Masaharu Tsubokura, Arinobu Hori, Yoshifumi Hayashi, Sotheara Chhim

**Affiliations:** ^1^Department of Psychiatry, Koh Kong Provincial Hospital, Koh Kong Province, Cambodia; ^2^Department of Public Health, Fukushima Medical University School of Medicine, Fukushima City, Fukushima 960 –1295, Japan; ^3^Sunrise Japan Hospital Phnom Penh, Khan Chroy Changvar Phnom Penh, Cambodia; ^4^Hori Mental Clinic, Minami Souma City, Fukushima 979-2335, Japan; ^5^Transcultural Psychosocial Organization Cambodia, Phnom Penh, Cambodia

## Abstract

In low- and middle-income countries in Asia, the use of supernatural, religious, and magical approaches to mental illness is widespread. We aimed to document the help-seeking behaviours and barriers to effective mental healthcare in the case of a psychiatric patient in rural Cambodia. The present case report describes the pathway that a patient with schizophrenia utilised to receive effective treatment in a rural area. First, the patient was taken by his parents to a pagoda. Subsequently, they took him to the home of a Kru Khmer (a Cambodian traditional healer). Nevertheless, his condition did not improve, and after seeing this, a neighbour suggested to his mother that they visit the provincial hospital. The patient received a diagnosis after an assessment by the hospital psychiatrist. Following several months of treatment with medication, the patient no longer exhibited paranoid behaviour. In this case, the patient's and his family's beliefs are strongly related to help-seeking behaviour toward medical care among psychiatric patients. To promote timely visits to the hospital, it is crucial to clarify and understand the type of beliefs held by psychiatric patients and their families. Besides, an educational approach to the beliefs is essential for shortening the duration of untreated illness.

## 1. Introduction

The WHO reports that at least 10% of the global adult population has mental and behavioural disorders [1]. Additionally, the public and economic burden of mental health disorders increased by 41% from 1990 to 2010 [2]. Action is required to reduce the suffering caused by mental disorders. Nevertheless, measures to address mental health burden are not progressing, especially in low middle-income countries (LMICs) and low-income countries (LICs), because of indifference and stigma towards mental disorders, lack of education about mental disorders and their treatment, lack of workforce engagement with mental health services, and extremely low mental health service funding [3]. Moreover, among the populations in Asian LMICs and LICs, there are severe deficiencies in the knowledge about mental disorders and their treatment, specifically regarding help-seeking options, available treatments, and consequences for help seeking [4]. To reduce cases of mental disorders as well as their social impact, it is necessary to alleviate patients' suffering using medical treatment. Thus, educating the population about mental disorders, particularly regarding help-seeking options and available treatments as well as the consequences for help seeking, is a critical public health issue.

In Asian LMICs and LICs, a major barrier to educating people about help-seeking behaviours and mental disorders is that, oftentimes, they are skeptical regarding mental healthcare [4]. Consequently, the use of supernatural, religious, and magical approaches to mental illness is prevalent [4–6]. Furthermore, employing these approaches can delay professional mental healthcare interventions when, ideally, people experiencing the symptoms of a mental disorder should be taken for treatment to a medical facility as soon as possible. Promoting public awareness on mental illness and effective treatments as well as dispelling myths could shorten or eliminate the delay between the onset of symptoms and professional mental healthcare intervention. Thus, it is crucial to identify the pathways that prospective patients use to seek treatment.

Cambodia is located in Southeast Asia and is classified as an LMIC by the World Bank [7]. The effort to address mental illness in Cambodia is changing from that of a trauma-focused mental health service in a postconflict state to a mental health service treating general mental illness [8]. However, in Cambodia, the number of psychiatrists is currently 0.35 per 100,000 population, which is much lower than the world average of 9 per 100,000 population [2, 9, 10]. Furthermore, the number of beds for inpatients is only 0.10 beds per 100,000 population, which is a limited number compared to the average in LMICs and LICs globally—7 beds per 100,000 population [9, 10]. Therefore, the government has decided to strengthen its mental health services [11]. Patient education about help-seeking behaviours is essential for advancing the delivery of mental health services. It is crucial to identify the pathways through which psychiatric patients seek treatment in order to educate them about help-seeking behaviours. Nevertheless, there is little information in the existing literature about the help-seeking behaviours of mental health patients in Cambodia [12].

This case report describes the pathway by which a psychiatric patient sought treatment in rural Cambodia. This case, which is similar to many others in Cambodian rural areas, is reported in the hopes of finding more effective solutions to providing mental health services. Informed consent was obtained from the patient's family.

## 2. Case Presentation

The subject of this report was a 28-year-old male patient with schizophrenia living in Koh Kong, a remote southwestern province of Cambodia. The man did not have other health problems. The onset of his symptoms began at the end of 2015; initially, the symptoms included poor sleep, headache, and irritability. He subsequently started to exhibit strange behaviours such as talking to himself, laughing or smiling without any reason, and whispering while alone. The subject started to become increasingly aggressive and began to hit his family and neighbours without provocation, claiming that they were cursing him. His parents took him to a pagoda which is a temple for Buddhist because they believed that his paranoid behaviour indicated possession by demons that could be driven off by the sacred power of monks. At the pagoda, a monk performed a ritual in which he hit the patient's body with a wooden stick and cleaned him with holy water. The family returned home after his treatment at the pagoda but found that his condition had not improved. His parents later took him to a Kru Khmer (Cambodian traditional healer) who they believed could heal their son by changing his fortune. The Kru Khmer prescribed traditional medicine, but the patient's condition remained unchanged.

Therefore, the family decided to lock up their son in a room to prevent him from hitting anyone.

On the recommendation of a neighbour, the family brought the patient to Koh Kong Provincial Hospital on 14 September 2016. The neighbour had informed his mother that the hospital had a psychiatrist on staff who could treat her son's illness. Koh Kong Provincial Hospital is a general public hospital with an outpatient department that provides psychiatric treatment to approximately 40–90 psychiatric outpatients per month.

When admitted to Koh Kong Provincial Hospital, the patient's hand was bound with a scarf and he was dressed poorly and had poor personal hygiene. He appeared older than his actual age. Despite being able to sit still, he was irritable and claimed that he was not ill. His thoughts were illogical; he could not orient himself to time, place, people, and was still having hallucinations.

After an assessment by the psychiatrist, the patient was prescribed haloperidol 5 mg, Artane 2.5 mg, and diazepam 2.5 mg per day for two weeks. The psychiatrist talked to his family about the diagnosis and method of treatment, and additionally, educated them on how to take the medications, their side effects, and how to deal with the patient's symptoms. The psychiatrist set a follow-up appointment two weeks later. Taking medication regularly under the family's careful attention improved the patient's condition. After several months of treatment with medication, the patient no longer exhibited paranoid behaviour and began to talk normally with his parents. Subsequently, his parents visited the hospital regularly to obtain long-term medication on his behalf. Long-term treatment was necessary for this patient; thus, the psychiatrist ensured that he and his family understood the importance of the treatment. The patient continued to take medication under the strict supervision of his family. This allowed him to live independently without being locked up at home.

## 3. Discussion

This case study describes the help-seeking behaviour of a psychiatric patient and related challenges. The patient and his family first sought help from a monk at a pagoda, and subsequently, consulted with a traditional healer. When the patient's psychiatric symptoms did not improve, his family consulted a hospital after being advised to do so by their neighbour. The information provided by the doctor regarding the importance of drug treatment led to the patient's recovery.

Patients' and their family's beliefs are strongly related to help-seeking behaviour among psychiatric patients. In this case, the patient's family prioritized consultation at a pagoda and with a traditional healer, believing that his symptoms were associated with demons or misfortune. Other studies on mental health in Cambodia have also reported culture-specific beliefs that express anxiety as a wind-related illness or weakness of the heart [13]. These beliefs result in help-seeking behaviours such as traditional treatment (e.g., coining) or consulting cardiology departments in medical facilities when a patient feels anxiety. To promote timely visits to the hospital, it is crucial to clarify and understand the type of beliefs held by psychiatric patients and their families.

In Cambodia, help-seeking behaviours resulting from culture-specific beliefs might add to the duration of untreated illness. In the present case, the hospital consultation was the last treatment sought for the patient. In previous studies, it has been reported that the duration of untreated illness in Cambodia was four times longer than that of developed countries; the duration of psychosis was particularly longer [14]. For patients with schizophrenia, delaying treatment with medication complicates the improvement of symptoms. In a previous Argentinian study, it was reported that providing knowledge about mental illness dramatically reduced the duration of untreated illness [15]. Therefore, an educational approach to the beliefs of patients and their families are essential for shortening the duration of untreated illness.

## 4. Conclusion

This case study described the help-seeking behaviour of typical psychiatric patients in rural Cambodia. For shorter durations of untreated illness, understanding patient beliefs is essential for changing help-seeking behaviour among psychiatric patients. It is important to promote the dissemination of appropriate information about psychiatric disorders and support positive collaboration among various mental health occupations. Furthermore, it is crucial to simultaneously use low-dose long-term medication and rehabilitation (both of which are still uncommon in Cambodia) to treat psychiatric patients.
